# Predicting treatment resistance in people with a first-episode of psychosis using commonly recorded clinical information

**DOI:** 10.1192/j.eurpsy.2022.303

**Published:** 2022-09-01

**Authors:** E.F. Osimo, B. Perry, P. Mallikarjun, G. Murray, O. Howes, P. Jones, R. Upthegrove, G. Khandaker

**Affiliations:** 1 University of Cambridge, Dept Of Psychiatry, Cambridge, United Kingdom; 2 Imperial College London, Institute Of Clinical Sciences, London, United Kingdom; 3 University of Birmingham, Institute Of Clinical Sciences, Birmingham, United Kingdom; 4 King’s College London, Psychiatry, London, United Kingdom

**Keywords:** treatment resistant schizophrenia, biomarkers, First Episode Psychosis, risk prediction

## Abstract

**Introduction:**

23% of people experiencing a first episode of psychosis (FEP) develop treatment resistant schizophrenia (TRS). At present, there are no established methods to accurately identify who will develop TRS from baseline.

**Objectives:**

In this study we used patient data from three UK early intervention services (EIS) to investigate the predictive potential of routinely recorded sociodemographic, lifestyle and biological data at FEP baseline for the risk of TRS up to six years later.

**Methods:**

We developed two risk prediction algorithms to predict the risk of TRS at 2-8 years from FEP onset using commonly recorded information at baseline. Using the forced-entry method, we created a model including age, sex, ethnicity, triglycerides, alkaline phosphatase levels and lymphocyte counts. We also produced a machine-learning-based model, including an additional four variables. The models were developed using data from two and externally validated in another UK psychosis EIS.

**Results:**

The development samples included 785 patients, and 1,110 were included in the validation sample. The models discriminated TRS well at internal validation (forced-entry: C 0.70, 95%CI 0.63-0.76; LASSO: C 0.69, 95%CI 0.63-0.77). At external validation, discrimination performance attenuated (forced-entry: C 0.63, 0.58-0.69; LASSO: C 0.64, 0.58-0.69) but recovered for the forced entry model after recalibration and revision of the lymphocyte predictor (C: 0.67, 0.62-0.73).

**Conclusions:**

The use of commonly recorded clinical information including biomarkers taken at FEP onset could help to predict TRS. These measures should be considered in future studies modelling psychiatric outcomes.

**Disclosure:**

No significant relationships.

